# A New MRI-Defined Biomarker for Rectal Mucinous Adenocarcinoma: Mucin Pool Patterns in Determining the Efficacy of Neoadjuvant Therapy

**DOI:** 10.3389/fonc.2020.01425

**Published:** 2020-08-20

**Authors:** Wuteng Cao, Lei Wu, Yandong Zhao, Jie Zhou, Wenli Li, Xinhua Wang, Jianbo Xu, Zhiyang Zhou, Changhong Liang

**Affiliations:** ^1^School of Medicine, South China University of Technology, Guangzhou, China; ^2^Department of Radiology, The Sixth Affiliated Hospital, Sun Yat-sen University, Guangdong Provincial Key Laboratory of Colorectal and Pelvic Floor Disease, Guangzhou, China; ^3^Department of Radiology, Guangdong Provincial People's Hospital, Guangdong Academy of Medical Sciences, Guangzhou, China; ^4^Department of Pathology, The Sixth Affiliated Hospital, Sun Yat-sen University, Guangdong Provincial Key Laboratory of Colorectal and Pelvic Floor Disease, Guangzhou, China; ^5^Guangzhou Universal Medical Imaging Diagnostic Center, Guangzhou, China

**Keywords:** rectal neoplasms, mucins, magnetic resonance imaging, neoadjuvant therapy, treatment outcome

## Abstract

**Background and Aim:** This work aims to study the relationship between MRI-defined mucin pool (MP) patterns prior to treatment and the efficacy of neoadjuvant therapy (NAT) in locally advanced rectal mucinous adenocarcinoma (RMAC).

**Methods:** This retrospective study included 278 RMAC patients evaluated between January 2012 and January 2019. After having been trained by using 118 cases with postoperative pathological images, radiologists distinguished MRI-defined MP status as mixed type (MTMP) and separate type (STMP) in a NAT cohort (160 patients) in addition to tumor characteristics, invasion of mesorectal facia, and nodal status. Reader reproducibility was determined using the κ coefficient. The main outcome was the accuracy of MP dichotomy in predicting whether patients had tumor responsiveness or not.

**Results:** Among 278 cases, MTMP and STMP accounted for 49.6 and 50.4% of MPs, respectively. A total of 72 patients received neoadjuvant chemoradiotherapy and 88 received chemotherapy. The tumor responsiveness rate in the chemoradiotherapy group was higher than that in the chemotherapy group (58.3 vs. 21.6%, *P* < 0.001). In the chemotherapy group, the tumor responsiveness rate in patients with MTMPs was lower than that in patients with STMPs (4.9 vs. 25.5%, *P* = 0.002). The baseline MRI-defined MTMP was associated with lower responsiveness rates after NAT in the chemotherapy group (odds ratio, 11.050, with 95% CI, 2.368–51.571, *P* = 0.002).

**Conclusions:** MP dichotomy can be reliably evaluated by using MRI. In the chemotherapy group, MTMP may be a dependent predictor to indicate a lower likelihood of tumor responsiveness after NAT.

## Introduction

Neoadjuvant therapy (NAT) followed by total mesorectal excision (TME) is the standard treatment for patients with locally advanced rectal cancer (1). Rectal mucinous adenocarcinoma (RMAC) is a subtype of rectal cancer comprising about 6.2–12.3% of cases (2, 3). Compared with non-mucinous adenocarcinoma, RMAC is much less sensitive to NAT (4, 5). Whether there is a need for individualized RMAC treatment options remains controversial (2, 6–8), and thus predicting the difference in curative effect in advance can provide a basis for the selection of neoadjuvant options.

The mucin pool (MP) has become a recent focus of research, and many investigators are currently exploring whether MP baseline characteristics are related to NAT efficacy (9–11). Magnetic resonance imaging (MRI), the recommended non-invasive tool in the guidelines, is often used to evaluate rectal cancer due to the high resolution of soft tissue. Additionally, T2-weighted imaging (T2WI) can be used to effectively identify the MP, which has a high T2WI signal (9, 12).

Some investigators showed that the MP in RMACs is a key factor affecting the NAT efficacy (11, 13), and Cao et al. found that when the proportion of the MP is >62.1%, the tumor burden is relatively low, and this could be used as an independent factor in predicting the efficacy of NAT (14). However, due to the MR image layer thickness, reconstruction of the three-dimensional volume is complicated and difficult in clinical practice.

Previous studies have focused on quantifying the MP (11, 13, 14), but there have been no reported studies on MP distribution. In clinical practice, we found a characteristic MP distribution. In some patients, the mucin lakes occur in large slices, and in others the MP components are intermingled with solid components of tumor. Whether the MP distribution type is related to the efficacy of NAT is unknown.

We hypothesized that MRI-defined MP classification prior to treatment could predict NAT efficacy. Here we used the baseline MRI T2WI to distinguish the MP type and, combined with other tumor characteristics, to determine whether baseline MRI-defined MP type could be used as a predictor of NAT efficacy.

## Materials and Methods

### Patients

This retrospective study was approved by the Ethics Committee of the Sixth Affiliated Hospital, Sun Yat-sen University. The need for informed patient consent was waived due to the retrospective nature of this study.

### Inclusion and Exclusion Criteria

The patient data were obtained between January 2012 and January 2019. The study included two cohorts, one for analyzing the consistency of MRI-defined MP type and pathological classification in the upfront surgery group without any neoadjuvant therapy (upfront surgery cohort) and the other for the NAT efficacy analysis (NAT cohort).

In the NAT cohort, the patients had a baseline MRI, had local advanced disease determined with baseline MRI (T3-4N_any_ or T1-2N1-2) and without any distal metastasis, had tumors with several high-signal mucus components (>50%) determined using T2WI (14, 15), had completed NAT with the regimen of modified FOLFOX6 with or without radiation (chemoradiotherapy or chemotherapy) according to FOWARC clinical trial (16, 17), had undergone TME after NAT, and had numerous postoperative pathological mucus lakes (>50%). Patients who had a history of other malignant tumors but did not complete NAT or had surgical resection, had insufficient MRI quality for evaluation, and had signet ring cell carcinoma after surgery were excluded.

In the upfront surgery cohort, the inclusion criteria were the same as those shown above, except for having NAT.

### MRI Acquisition

The MRIs of all patients were performed with a 1.5-T MR unit (Optimal 360, GE Healthcare, Waukesha, WI, USA). Without any bowel preparation, the patients were injected intramuscularly with 20 mg of scopolamine butylbromide 30 min prior to imaging to reduce colonic motility. The rectal MRI protocol included oblique axial, coronal, and sagittal T2-weighted images, oblique axial T1-weighted images, diffusion-weighted images, and gadolinium-enhanced T1-weighted images, as summarized in Table 1.

**Table 1:** The technical MRI parameters of the scanning sequences.

**Planes**	**TR/TE (ms)**	**NEX**	**FOV (cm)**	**Matrix**	**Section thickness/gap (mm)**
Oblique axial T2WI	4,294/108	4	24 × 24	288 × 256	3/0.5
Oblique coronal T2WI	2,358/108	4	24 × 24	288 × 256	3/0.5
Oblique sagittal T2WI	2,591/125	4	28 × 28	288 × 224	3/0.5
Oblique axial DWI (*b* = 0.1200 s/mm^2^)	4,550/92.6	4	40 × 40	192 × 192	3/0.5
Oblique axial T1WI	4,19/13.5	2	24 × 24	320 × 224	3/0.5

*TR, repetition time; TE, echo time; NEX, number of excitation; DWI, diffusion-weighted imaging; T1WI, T1-weighted imaging; T2WI, T2-weighted imaging*.

### Evaluation of MP Distribution Pattern

#### MRI-Defined MP Type Acquisition in the Upfront Surgery Cohort Patients

The comparison of preoperative MRI-defined MP type with postoperative pathological MP was performed in 118 patients with RMAC who underwent direct surgery without NAT. The preoperative T2WI was used to identify the MP (distinctly high signal) and tumor solid components (medium signal) (14). According to the distribution characteristics of MP, two radiologists (readers 1# and 2#, with 8 and 20 years of gastrointestinal diagnosis experience, respectively) independently divided all patients into two types: the mixed type (MTMP), which showed mucus components containing abundant solid tumor components, and the separate type (STMP), which showed secreted mucus components outside the solid tumor area. The inter-reader agreement between the two radiologists and the intra-reader consistency of the first radiologist were assessed.

#### Pathologic MP Type Acquisition in the Upfront Surgery Cohort Patients

Two pathologists (6 and 20 years of experience in pathological diagnosis of gastrointestinal lesions, respectively) extracted hematoxylin–eosin (H&E)-stained sections from the specimens for reanalysis. The H&E staining images can be one of three tumor types: pathological mixed type (pMTMP), separate type (pSTMP), and undeterminable type. The pMTMP was defined as the interstitial composition of the tumor solid and MP. The pSTMP was defined as a large number of dense tumor cells, and the MP was relatively independent. Some patients could not be classified and were identified as undeterminable type. The first pathologist performed the reanalysis after 6 months. The consistency between two reviewers and between the same reviewer was evaluated, respectively.

#### MRI-Defined MP Type Training After Comparison to the Pathologic MP Type

**Figure 1:**
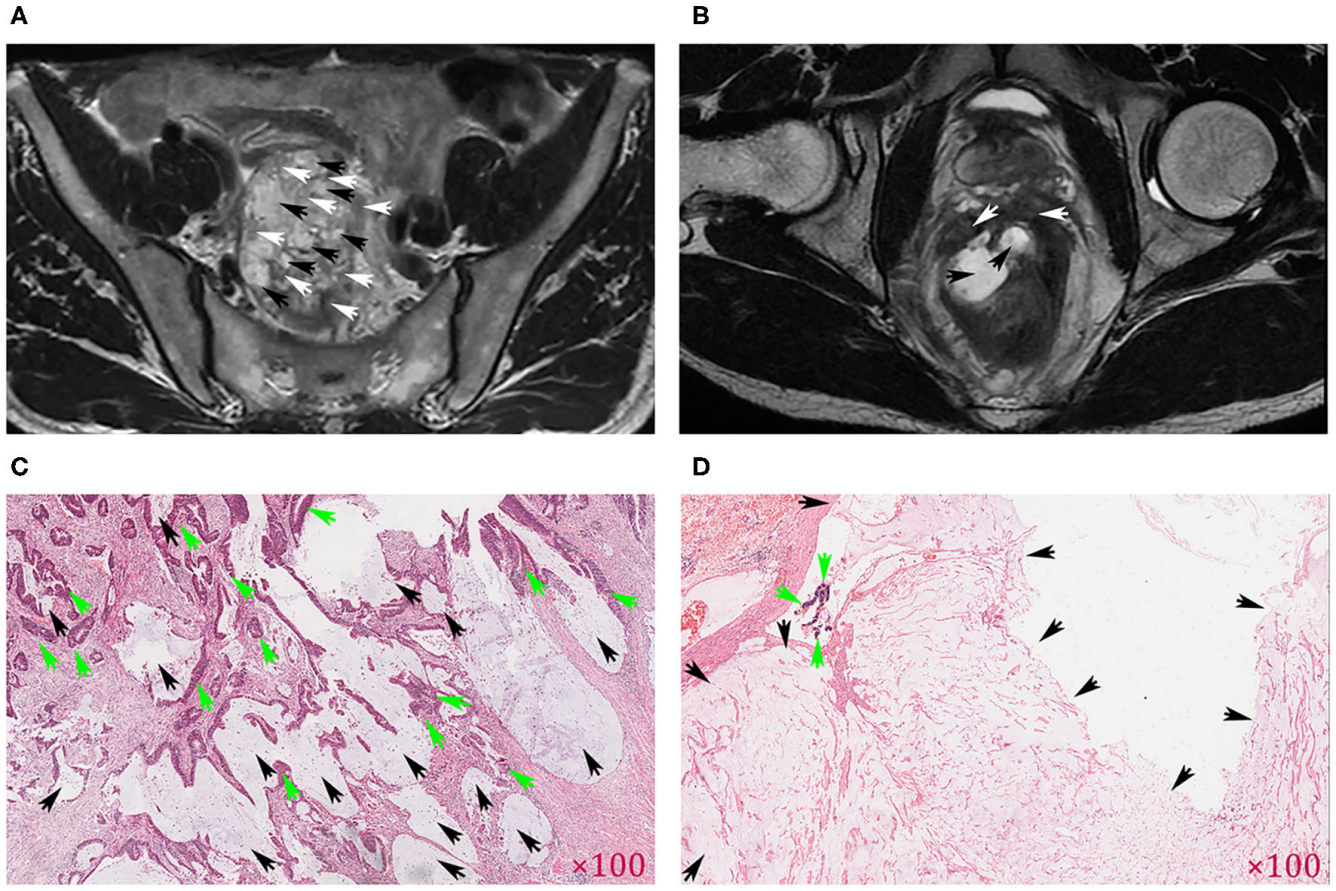
Comparison of MR images of the mucin pool (MP) and pathological images. The axial T2-weighted images indicate that the mucin pool (black arrows) was mixed with the tumor tissue (white arrows) and was identified as MRI-defined mixed-type MP (MTMP) **(A)**. If the mucin pool (black arrow) produced a high signal (higher than that of mesorectal fat), the tumor component (white arrow) was limited to the rectal wall, and the mucin pool was relatively independent, defined as MRI-defined separate-type MP (STMP) **(B)**. **(C)** Clustered and flaky solid tumor components (green arrows) were mixed with the mucin pool (black arrows), consistent with MRI-defined MTMP in **(A)**. **(D)** Large patches of mucin pool (black arrows) exist independently, consistent with MRI-defined STMP in **(B)**. Original magnification of H&E-stained tissue section in **(C,D)**.

The results of the MRI-defined MP classifications and the pathological MP classifications were compared (Figure 1). For patients with inconsistent MRI-defined MP types and pathological MP types, a radiologist (reader #, with 30 years of gastrointestinal diagnosis experience) convened all radiologists and pathologists to discuss and form a consensus on the binary classification of MRI-defined MP distribution characteristics (Table 2).

**Table 2:** MRI-defined and pathological MP type.

**MP pattern**	**MRI-defined MP type**			**Pathology-defined MP type**
	**MP SI on T2WI**	**Solid tumor component SI on T2WI**	**MP relationship with solid tumor component**	
MTMP	Significantly higher than the muscularis propria, lower than the mesorectal fat	Equal to or slightly above the muscularis propria	MP and solid components are cross-distributed	The solid components of the tumor are clustered or flaky, and the two components are cross-distributed. The mucus lake is floating in the tumor components
STMP	Significantly high signal (equal to or higher than the mesorectal fat)	Equal to or slightly above the muscularis propria	MP is not mixed with solid component	A large number of mucus lakes, in which clustered or flaky tumor components cannot be found, but a few tumor cells can be found scattered within the MP. The solid components of the tumor are relatively limited

*SI, signal intensity; MP, mucin pool; MTMP, mixed-type MP; STMP, separate-type MP*.

#### Baseline MRI-Defined MP Type Evaluation in the NAT Cohort

After the training described above, the two radiologists distinguished the MRI-defined MP status as MTMP and STMP. The inter-reader agreement between the two radiologists and the intra-reader consistency of the first radiologist were assessed. If a κ coefficient larger than 0.60 was obtained, then the initial evaluation was used for the following analysis.

### Evaluation of Baseline MRI-Defined Tumor Features

The baseline tumor MRI image characteristics were independently assessed by a radiologist (#), with 8 years of experience in gastrointestinal diagnostics. The characteristics evaluated included tumor maximal length (TML), distance from the inferior part of the tumor to the anal verge (DTA), mesorectal fascia (MRF), tumor infiltration (T staging), and lymph node status (N staging). A total of 30 patients were randomly selected to be reevaluated by another radiologist (with 5 years of experience in gastrointestinal imaging) in order to verify the inter-reader agreement.

### Criteria for Tumor Responsiveness

The surgically resected specimens in NAT cohort were pathologically analyzed according to the seventh edition of the American Joint Committee on Cancer TNM staging system (18) and Ryan et al. (19). The pathologic assessment of tumor staging (T0–T4b), lymph node staging (N0–N2), and pathologic tumor regression grade (TRG) was collected. Complete response was defined as the absence of viable tumor cells in the primary tumor and lymph nodes. All patients were categorized by the therapy response based on the TRG: a responsive group (TRG 0 with no viable cancer cells or TRG 1 with single cells) and a non-responsive group (TRG 2 with residual cancer outgrown by fibrosis or TRG 3 with fibrosis outgrown by residual cancer or without fibrosis but with extensive residual cancer) (14, 15).

### Statistical Analysis

This study aimed to explore the prognostic effect of MRI-defined MP type on tumor responsiveness after neoadjuvant therapy. First, kappa test was used to assess the intra- or inter-reader agreement of categorical variables (e.g., MRI-defined MP classification, MRF, T and N staging), and the concordance was classified as follows: poor (0–0.20), fair (0.21–0.40), moderate (0.41–0.60), substantial (0.61–0.80), or perfect (0.81–1.00). The interobserver agreement of continuous variables was evaluated by intra-class correlation coefficient (ICC) (e.g., DTA and TML), and ICC >0.75 was considered as good agreement. Second, chi-square test was used to assess the difference of categorical variables, such as MRI-defined MP type and the tumor responsiveness of NAT. Third, stepwise logistic regression model was used for multivariate analysis by selecting independent prognostic factors for tumor responsiveness from all baseline MRI features and to acquire the odds ratios (OR). The independent prognostic factors were used to group the patients into different risk stratifications, and the rate of tumor responsiveness was presented in a nomogram with scores for each stratified population.

Calculations were performed with SPSS (version 22.0; SPSS, Chicago, IL, USA). *P* < 0.05 were considered as statistically significant.

## Results

**Figure 2:**
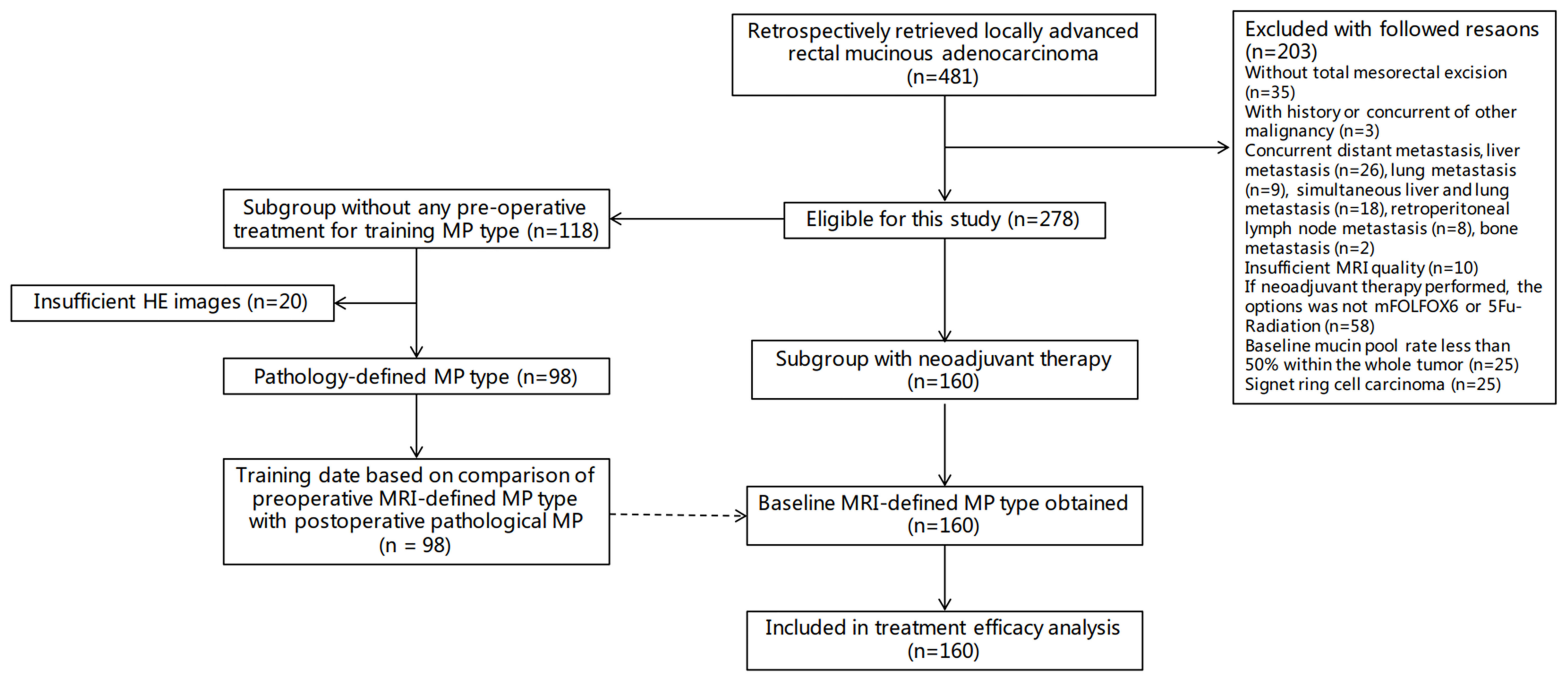
Study flowchart. MP, mucin pool.

Figure 2 shows the study flowchart. Among 278 patients with rectal mucinous adenocarcinoma, 185 (66.5%) were men and 93 (33.5%) were women, with an average age of 50 years (range, 27–83 years).

### Consistency Among Observers

In terms of MRI-defined MP of the upfront surgery cohort, the inter-reader agreement of the two radiologists achieved 0.779 [95% confidence interval (CI), 0.665–0.892]. The κ value of intra-reader agreement of the first radiologist was 0.898 (95% CI, 0.818–0.977) (Tables S1, S2). There were 20 patients with pathological section damage or poor image resolution. A total of 98 of 118 (83%) were classified into pathological MP types, with 55 being pathological MTMP and 43 as STMP. The κ coefficient of inter-reviewer agreement of the two pathologists achieved 0.876, with 95% CI of 0.781–0.972, and the κ value of intra-reviewer was 0.917 (95% CI, 0.838–0.997) (Tables S3, S4). As to the consistency between MRI-defined and pathology-defined MP, the κ coefficient was 0.773 (95% CI, 0.646–0.899) (Table S5).

After training, the inter-reader agreement of the two radiologists achieved 0.849 (95% CI, 0.766–0.931), and the intra-reader agreement of the first radiologist achieved 0.950 (95% CI, 0.901–0.998) (Tables S6, S7).

Additionally, in terms of baseline MRI-defined T and N staging, the ICC of inter-reader agreement of the two radiologists achieved 0.746 (95% CI, 0.552–0.863) and 0.718 (95% CI, 0.492–0.854), respectively. As to the DTA and TML measurements, the ICC was 0.982 (95% CI, 0.941–0.991) and 0.707 (95% CI, 0.483–0.844), respectively. As to the inter-reader agreement of the two radiologists for evaluating MRF, the κ value was 0.792 (95% CI, 0.515–1.000).

### Correlation Between Baseline MRI-Defined MP Type and the Tumor Characteristics in the NAT Cohort

Seventy-three patients with MRI-defined MTMP and 87 STMP were identified. There was no statistically significant difference in the distribution of NAT regimen (mFOLFOX6 with or without radiation) between the two MP types (*P* = 0.787). In patients with MTMP and STMP, the ratio of T4b based on baseline MRI was 21.9% (16/73) and 10.3% (9/87), respectively, and the positive lymph node rate was 68.5% (50/73) and 69.0% (60/87), respectively. There were no statistically significant differences in T staging, MRF status, N staging, DTA, TML, BMI, CEA, age, and sex between the STMP and the MTMP patients (Table 3).

**Table 3:** Tumor characteristics between two mucin pool patterns.

**Characteristic**	**Number of patients**	**MRI-defined MTMP (*n* = 73)**	**MRI-defined STMP (*n* = 87)**	***P Value***
Age (years)				0.852
<51	78	35 (47.9)	43 (49.4)	–
≥51	82	38 (52.1)	44 (50.6)	–
Sex				0.536
Man	110	52 (71.2)	58 (66.7)	–
Women	50	21 (28.8)	29 (33.3)	–
CEA (ng/ml)				0.294
<5	87	43 (58.9)	44 (50.6)	–
≥5	73	30 (41.1)	43 (49.4)	–
BMI (kg/m^2^)				0.135
<22	87	39 (53.4)	48 (55.2)	–
≥22	73	34 (46.6)	39 (44.8)	–
Baseline MRF Status				0.111
Positive	45	16 (21.9)	29 (33.3)	–
Negative	115	57 (78.1)	58 (66.7)	–
DTA (cm)				0.811
<5	83	38 (52.1)	45 (51.7)	–
5–10	57	24 (32.9)	33 (37.9)	–
≥10	20	11 (15.1)	9 (10.3)	–
TML (cm)				0.494
<5	88	38 (52.1)	50 (57.5)	–
≥5	72	35 (47.9)	37 (42.5)	–
Baseline T staging				0.211
1–2	11	4 (5.5)	7 (8.0)	–
3	106	47 (64.4)	59 (67.8)	–
4a	18	6 (8.2)	12 (13.8)	–
4b	25	16 (21.9)	9 (10.3)	–
Baseline N staging				0.942
0	50	23 (31.5)	27 (31.0)	–
1	46	21 (28.8)	25 (28.7)	–
2	64	29 (39.7)	35 (40.2)	–
Pathological T staging				0.114
0	20	8 (11.0)	12 (13.8)	–
1	9	6 (8.2)	3 (3.4)	–
2	13	5 (6.8)	8 (9.2)	–
3	96	51 (69.9)	45 (51.7)	–
4a	16	3 (4.1)	13 (14.9)	–
4b	6	0 (0)	6 (6.9)	–
Pathological N staging				0.245
0	82	40 (54.8)	42 (48.3)	–
1	41	20 (27.4)	21 (24.1)	–
2	37	13 (17.8)	24 (27.6)	–
TRG				0.009
0	20	8 (11.0)	12 (13.8)	–
1	41	12 (16.4)	29 (33.3)	–
2	74	37 (50.7)	37 (42.5)	–
3	25	16 (21.9)	9 (10.3)	–
NAT regimen				0.787
Without radiation	88	41 (56.2)	47 (54.0)	–
With radiation	72	32 (43.8)	40 (46.0)	–

*Data are number of patients, with percentages in parentheses*.

*MRF, mesorectal fascia; DTA, distance from the inferior part of the tumor to the anal verge; TML, tumor maximal length; BMI, body mass index; MTMP, mixed type of mucin pool; STMP, separate type of mucin pool; NAT, neoadjuvant therapy; TRG, tumor regression grade*.

### Relationship of Baseline MRI-Defined MP Type With Tumor Responsiveness Rate

Among the 160 patients, the rate of tumor responsiveness after NAT was 38.1% (61/160) and the rate of non-responsiveness was 61.9% (99/160). The responsiveness rate in the MRI-defined MTMP group was 27.4%, lower than that in the STMP group at 47.1% (*P* = 0.011) (Table 4). There was no statistical difference in CEA, MRF status, DTA, BMI, sex, T staging, and N staging between the responsiveness and the non-responsiveness groups. However, the responsiveness rate of RMAC in the neoadjuvant chemoradiotherapy group was higher than that in the chemotherapy group (*P* < 0.001).

**Table 4:** Association between baseline MRI-defined mucin pool pattern, tumor characteristics, and tumor responder.

**Characteristic**	**Number of patients**	**Responsiveness group (*n* = 61)**	**Non-responsiveness group (*n* = 99)**	***P Value***
Age(years)				0.932
<51	78	30 (49.2)	48 (48.5)	–
≥51	82	31 (50.8)	51 (51.5)	–
Sex				0.743
Man	110	41 (67.2)	69 (69.7)	–
Women	50	20 (32.8)	30 (30.3)	–
CEA (ng/ml)				0.480
<5	87	31 (50.8)	56 (56.6)	–
≥5	73	30 (49.2)	43 (43.4)	–
Baseline MRF status				0.436
Positive	45	15 (24.6)	30 (30.3)	–
Negative	115	46 (75.4)	69 (69.7)	–
DTA status (cm)				0.368
<5	83	28 (45.9)	55 (55.6)	–
5–10	57	26 (42.6)	31 (31.3)	–
≥10	20	7 (11.5)	13 (13.1)	–
TML (cm)				0.406
<5	88	31 (50.8)	57 (57.6)	–
≥5	72	30 (49.2)	42 (42.4)	–
BMI (kg/m^2^)				0.302
<22	87	30 (49.2)	57 (57.6)	–
≥22	73	31 (50.8)	42 (42.4)	–
Baseline T staging				0.161
1–2	11	5 (8.2)	6 (6.0)	–
3	106	35 (57.4)	71 (71.7)	–
4a	18	7 (11.5)	11 (11.1)	–
4b	25	14 (23.0)	11 (11.1)	–
Baseline N staging				0.753
0	50	18 (29.5)	32 (32.3)	–
1	46	18 (29.5)	28 (28.3)	–
2	64	25 (41.0)	39 (39.4)	–
MRI-defined MP type				0.011
MTMP	73	20 (32.8)	53 (53.5)	–
STMP	87	41 (67.2)	46 (46.5)	–
NAT regimen				<0.001
Without radiation	88	19 (31.1)	69 (69.7)	–
With radiation	72	42 (68.9)	30 (30.3)	–

*Data are number of patients, with percentages in parentheses*.

*MRF, mesorectal fascia; DTA, distance from inferior part of tumor to the anal verge; TML, tumor maximal length; BMI, body mass index; MTMP, mixed type of mucin pool; STMP, separate type of mucin pool; NAT, neoadjuvant therapy*.

**Figure 3:**
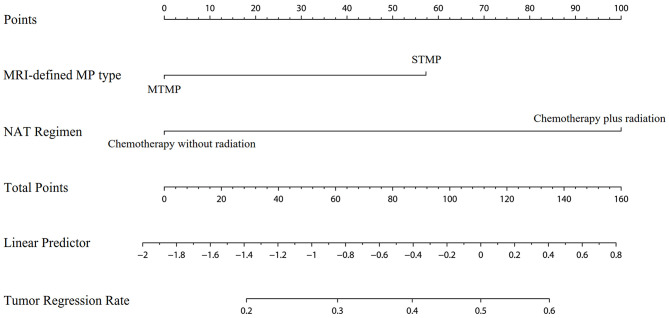
Nomogram for the prediction of tumor responsiveness based on baseline MRI-defined mucin pool type and neoadjuvant therapy regimen.

A logistic regression analysis identified the baseline MRI-defined MP type and NAT regimen as independent predictors. Patients with STMPs had a higher tumor responsiveness rate than patients with MTMPs, with an OR of 2.637 (95% CI, 1.274–5.435; *P* = 0.009). The tumor responsiveness rate of the chemoradiotherapy group was higher than that of the chemotherapy group, and the OR was 5.415 (95% CI, 2.649–11.070; *P* < 0.001). The model that incorporated the above-mentioned two predictors was developed and presented as the nomogram (Figure 3).

### Independent Predictor (MTMP) of Lower Tumor Responsiveness in the Chemotherapy Group

In the mFOLFOX6 group, only two patients with MTMPs developed tumor responsiveness after NAT, a significantly lower rate (4.9%) than that of the STMP patients (25.5%) (*P* = 0.002). As to other baseline MRI-defined features, there were no statistically significant differences in CEA, MRF status, DTA, BMI, sex, T staging, and N staging between the responsive and the non-responsive patients. In univariate analysis, MTMP was the factor leading to low tumor responsiveness in the chemotherapy group. The OR of STMP was 11.050 (2.368–51.571, *P* = 0.002).

In the chemoradiotherapy group (mFOLFOX6 plus radiation), there was no statistically significant difference in the MRI-defined MP type between the responsiveness and the non-responsiveness groups, with a responsiveness rate of 56.3 and 60.0% in STMP and MTMP patients, respectively (*P* = 0.748).

## Discussion

In the neoadjuvant chemotherapy group, baseline MRI-defined MTMP was associated with a lower likelihood of tumor responsiveness, with a rate of 4.9% in RMAC patients. In terms of tumor responsiveness, the OR of MRI-defined STMP to MTMP was 11.050.

Among the RMAC patients, 15.6% of them had invasion of adjacent organs and structures (cT4b) at baseline. Most of the patients were at T3 or greater stages, indicating that tumors often break through the muscularis propria (5, 7, 20). In our study, 21.9% of the patients with MTMPs were evaluated by MRI as having tumors involving adjacent organs before NAT, while all of those patients were confirmed as non-pT4b (with spared organs) by pathology. This suggests that these patients may have no tumor cells in the mucus pool adjacent to the surrounding organs after NAT. We hypothesized that patients with MTMPs resembled conventional non-mucinous adenocarcinoma, and after NAT, the tumor regressed and the volume decreased significantly (21). However, for patients with STMPs, the mucin pool is often distributed outside the rectal wall in surrounding organs, and the volume of this mucin component is not significantly reduced after NAT. Therefore, it is a challenge to judge whether tumor-active cells remain in the organ-invasive mucus components after NAT.

The mFOLFOX6 regimens, with or without radiation, are two options according to the studies of Deng et al. (16, 17). Some studies showed that radiotherapy may increase the risk of anastomotic leakage and cause anal, urinary, and sexual dysfunction (22, 23). There are no significant differences in the rate of surgical margin, permanent colostomy, and overall survival between neoadjuvant chemotherapy and radiotherapy (3, 24, 25). However, some studies indicated that radiotherapy can better control local recurrence (26, 27). In this study, the tumor responsiveness rate of RMAC patients with chemoradiotherapy was 58.3%, which was significantly higher than that of the regimen without radiation. This finding suggests that, for RMAC, radiation should be strongly considered because it can cause a higher rate of tumor responsiveness, while chemotherapy alone has a lower responsiveness rate. Radiotherapy can cause changes in MP, and the reduction of tumor components in mucin lakes could be behind the tumor responsiveness (10, 11).

In the chemotherapy group, the tumor responsiveness rate of patients with MTMP was extremely low (4.9%), which was significantly lower than that of rectal non-mucinous adenocarcinoma reported in the literature (7, 28). This suggests that the mucin lakes at baseline play a key role in therapeutic response and that it is often difficult to achieve tumor responsiveness by mFOLFOX6 without radiation. In comparison, patients with STMPs are more likely to have tumor responsiveness with a response rate of 25.5%. In the tumors of patients with STMPs, T2WI shows a large number of high-signal regions, while the solid tumor components (medium signal) are relatively small. Thus, we speculate that the mucus pool may account for a higher proportion of overall tumor volume in patients with STMPs due to mucus hypersecretion. Similar to previous studies, patients with high mucin components and few solid components are more likely to have tumor responsiveness (9, 10, 14).

The MRI-defined MP dichotomy is a non-invasive tool used to reflect tumor responsiveness after NAT. Our results suggest that different distribution types of mucus pool may have different sensitivities to NAT, and particularly, the MTMP with a low responsiveness rate needs to be separated from the STMP to provide guidance for the clinical multidisciplinary team. Park *et al*. showed that the use of a MRI-defined TRG system can predict the pathological TRG of NAT for RMAC patients (9), but the issue of predicting the presence or the absence of tumor cells in mucus components that are closely related to the depth of tumor infiltration has not been resolved. Thus, since the presence of active tumor components in mucus pools cannot be determined by MRI, MRI-defined MP distribution type may be a new biomarker to reflect the tumor response after NAT.

According to the nomogram of our study, we speculate that patients with locally advanced RMAC with MRI-defined MTMP type have difficulty in achieving tumor responsiveness after NAT. When patients with locally advanced RMAC choose NAT, the chemotherapy regimen (without radiation) may not be appropriate for RMAC. Therefore, patients with RMAC with baseline MRI-defined MTMP may benefit from radiation in addition to neoadjuvant chemotherapy.

One of the innovations lies in the use of upfront surgery patients to verify the accuracy of MRI-defined MP classification, which refers to postoperative pathological images (29). We did not adopt the traditional training method of one-to-one correspondence between MRI and the pathological images of some patients. We think that MRI can be used instead to distinguish the two distributions of mucus components based on experience gained from clinical work, so the mucus pool was directly classified by using MRI. After comparing MRI-defined MP type with pathological features, it was found that the accuracy of MRI classification reached 88.8% in this study. Then, the cases with inconsistent MRI and pathology classifications were discussed to further enhance the radiologist's experience in identifying MP distribution and form a trained protocol. Finally, this MRI-defined protocol was used to evaluate the baseline MRI-defined MP of patients in the NAT group prior to surgery.

Several limitations deserve mention. Firstly, this study was a single-center retrospective analysis. Secondly, the treatment strategy of the enrolled patients was based on the FOWARC study (16, 17), which were mFOLFOX6 or mFOLFOX6-based chemoradiotherapy. Thus, the efficacy of other neoadjuvant regimens in patients with RMAC is not clear. Thirdly, the endpoint of this study was tumor responsiveness after NAT. At a median follow-up of 23 months (range 2–85 months), the baseline MRI-defined MP type was not associated with overall survival or disease-free survival (Figure S1). Some literature shows that the long-term survival of RMAC patients can benefit from NAT, but due to insufficient follow-up time, the difference in long-term survival between patients of MTMP and STMP still requires further research. Finally, the use of MRI can cause some signet ring cell carcinomas (SRCC) with mucus components to be easily confused with RMAC. Thus, we used a retrospective analysis of pathological data in the inclusion criteria to exclude the subcategory of SRCC as this carcinoma had no significant response to NAT than RMAC (29). The predictive application of MRI-defined MP binary classification scheme in SRCC needs to be carried out in the next research.

In conclusion, this study demonstrates that baseline MRI-defined MP type can be reliably identified and can serve as an independent predictor for tumor regression in patients with locally advanced RMAC. Patients with MTMPs had very low rates of tumor responsiveness when receiving neoadjuvant chemotherapy without radiation. With further high-quality evidence, MRI-defined MP dichotomy should be included in routine baseline MRI reports to craft an individualized treatment strategy.

## Data Availability Statement

The raw data supporting the conclusions of this article will be made available by the authors, without undue reservation.

## Ethics Statement

The studies involving human participants were reviewed and approved by The Ethics Committee of the Sixth Affiliated Hospital, Sun Yat-sen University. Written informed consent for participation was not required for this study in accordance with the national legislation and the institutional requirements.

## Author Contributions

CL, ZZ, and WC contributed to the study conception and design. WC, LW, YZ, and WL contributed to the acquisition of data. WC, LW, JZ, XW, and WL analyzed and interpreted the data. WC, ZZ, and CL drafted the manuscript. ZZ and CL critically revised the manuscript. All authors contributed to the article and approved the submitted version.

## Conflict of Interest

The authors declare that the research was conducted in the absence of any commercial or financial relationships that could be construed as a potential conflict of interest.

## Supplementary Material

Figure S1 Kaplan–Meier estimates of overall survival **(A)** and disease-free survival **(B)** according to the baseline MRI-defined mucin pool type in the NAT cohort of patients (log-rank statistical test *P* = 0.880 and 0.852, respectively). NAT, neoadjuvant therapy.

## Abbreviations

RMAC: rectal mucinous adenocarcinoma
NAT: neoadjuvant therapy
MTMP: mixed type of mucin pool
STMP: separate type of mucin pool
CEA: carcino-embryonic antigen
BMI: body mass index
MRF: mesorectal fascia
TML: tumor maximal length
DTA: distance from inferior part of tumor to the anal verge
ROC: receiver operating characteristic.
