# Supplementary material for: A New MRI-Defined Biomarker for Rectal Mucinous Adenocarcinoma: Mucin Pool Patterns in Determining the Efficacy of Neoadjuvant Therapy
Source: Front Oncol. 2020 Aug 20;10:1425. doi: 10.3389/fonc.2020.01425 (PMC7468516; doi:10.3389/fonc.2020.01425)
Supplement: Supplementary file 1 [file Table_1.docx]

**Supporting Information Table 1-7**. MP classification and radiologic-pathologic correlation in training-cohort and NAT-cohort Date. MP = mucin pool, MTMP = mixed type of mucin pool, STMP = separate type of mucin pool, NAT = neoajuvant therpay

**Supporting Information Table 1.** MRI-defined MP type from preoperative MRI T2 weighted images evaluated by two radiologists in training-cohort

| Radiologist1 | Radiologist2 | | Total |
| --- | --- | --- | --- |
|  | MTMP | STMP |  |
| MTMP | 57 | 8 | 65 |
| STMP | 5 | 48 | 53 |
| Total | 62 | 56 | 118 |

With k coefficient, 0.779 (95% CI, 0.665-0.892)

**Supporting Information Table 2.** The inter-reader data of MRI-defined MP type from preoperative MRI T2 weighted images evaluated by the first radiologist in training-cohort

| Radiologist1 | Radiologist1-Repeat | | Total |
| --- | --- | --- | --- |
|  | MTMP | STMP |  |
| MTMP | 61 | 4 | 65 |
| STMP | 2 | 51 | 53 |
| Total | 63 | 55 | 118 |

With k coefficient, 0.898 (95% CI, 0.818-0.977)

**Supporting Information Table 3.** The MP type basing on pathology images evaluated by two pathologists in training-cohort

| Pathologist1 | Pathologist2 | | Total |
| --- | --- | --- | --- |
|  | pMTMP | pSTMP |  |
| pMTMP | 51 | 4 | 55 |
| pSTMP | 2 | 41 | 43 |
| Total | 53 | 45 | 98 |

With k coefficient, 0.876 (95%CI: 0.781-0.972)

**Supporting Information Table 4.** The MP type basing on pathology images evaluated by the first pathologist in training-cohort

| Pathologist1 | Pathologist1-Repeat | | Total |
| --- | --- | --- | --- |
|  | MTMP | STMP |  |
| MTMP | 53 | 2 | 55 |
| STMP | 2 | 41 | 43 |
| Total | 55 | 43 | 98 |

With k coefficient, 0.917 (95% CI, 0.838-0.997)

**Supporting Information Table 5.** The consistency between MRI-defined and pathology-defined MP for radiologist 1 and pathologist 1 in training-cohort

| Radiologist1 | Pathologist1 | | Total |
| --- | --- | --- | --- |
|  | MTMP | STMP |  |
| MTMP | 49 | 5 | 54 |
| STMP | 6 | 38 | 44 |
| Total | 55 | 43 | 98 |

With k coefficient, 0.773 (95% CI, 06464-0.899)

**Supporting Information Table 6.** The baseline MRI-defined MP type evaluated by two trained radiologists in NAT-cohort

| Radiologist1 | Radiologist2 | | Total |
| --- | --- | --- | --- |
|  | Baseline MTMP | Baseline STMP |  |
| Baseline MTMP | 66 | 7 | 73 |
| Baseline STMP | 5 | 82 | 87 |
| Total | 71 | 89 | 160 |

With k coefficient, 0.849 (95% CI, 0.766-0.931)

**Supporting Information Table 7.** The baseline MRI-defined MP type evaluated by the first radiologist in NAT-cohort

| Radiologist1 | Radiologist1-Repeat | | Total |
| --- | --- | --- | --- |
|  | Baseline MTMP | Baseline STMP |  |
| Baseline MTMP | 72 | 1 | 73 |
| Baseline STMP | 3 | 84 | 87 |
| Total | 75 | 85 | 160 |

With k coefficient, 0.950 (95% CI, 0.901-0.998)
