# Supplementary figures and images for: A New MRI-Defined Biomarker for Rectal Mucinous Adenocarcinoma: Mucin Pool Patterns in Determining the Efficacy of Neoadjuvant Therapy
Source: Front Oncol. 2020 Aug 20;10:1425. doi: 10.3389/fonc.2020.01425 (PMC7468516; doi:10.3389/fonc.2020.01425)

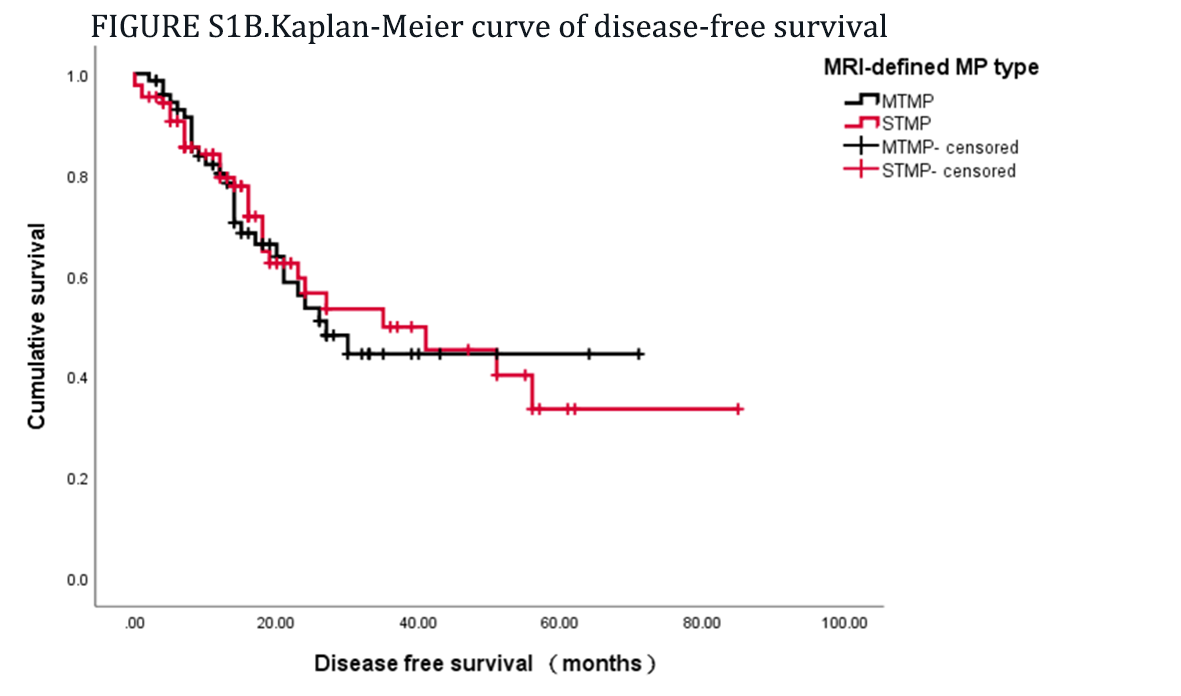

Supplement: Supplementary file 2 [file Image_2.tif]

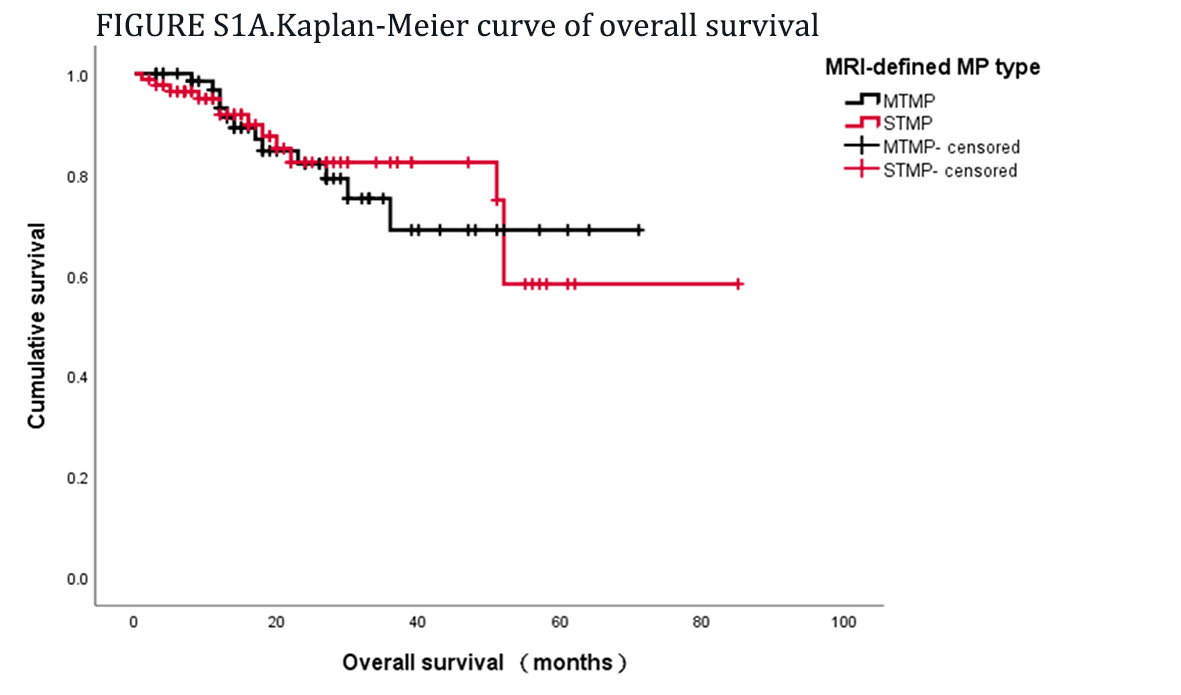

Supplement: Figure S1 — Kaplan–Meier estimates of overall survival (A) and disease-free survival (B) according to the baseline MRI-defined mucin pool type in the NAT cohort of patients (log-rank statistical test P = 0.880 and 0.852, respectively). NAT, neoadjuvant therapy. [file Image_1.tif]
